# Desmoid-Type Fibromatosis of the Proximal Jejunum Mimicking Gastric Gastrointestinal Stromal Tumor: Diagnostic Pitfalls in Determining the Tumor Origin

**DOI:** 10.70352/scrj.cr.26-0440

**Published:** 2026-10-01

**Authors:** Chiaki Ikeshita, Keiji Nishibeppu, Toshiyuki Kosuga, Shutaro Sumiyoshi, Hiroyuki Inoue, Kento Kurashima, Kazuya Takabatake, Hidemasa Kubo, Jun Kiuchi, Taisuke Imamura, Kenji Nanishi, Hiroki Shimizu, Tomohiro Arita, Yusuke Yamamoto, Hirotaka Konishi, Ryo Morimura, Hitoshi Fujiwara, Atsushi Shiozaki

**Affiliations:** Division of Digestive Surgery, Department of Surgery, Kyoto Prefectural University of Medicine, Kyoto, Kyoto, Japan

**Keywords:** desmoid-type fibromatosis, proximal jejunum, obstruction

## Abstract

**INTRODUCTION:**

Desmoid-type fibromatosis (DTF) of the small intestine is rare and often mimics gastrointestinal stromal tumor (GIST). Its infiltrative growth can also make identification of the true organ of origin difficult.

**CASE PRESENTATION:**

A 56-year-old woman was referred with a suspected gastric GIST based on imaging and endoscopic US-guided fine-needle aspiration. Before the planned surgery, the patient developed anorexia and vomiting. Emergency CT revealed rapid tumor enlargement with duodenal obstruction. Urgent multi-organ resection was performed, including partial gastrectomy, jejunal resection, partial pancreatic resection, and partial colectomy. Intraoperatively, the tumor appeared to originate from the stomach; however, histopathological examination revealed DTF arising from the jejunum with secondary invasion into adjacent organs. The postoperative course was complicated by an intra-abdominal abscess, which was successfully managed. No recurrence was observed 12 months after surgery.

**CONCLUSIONS:**

DTF should be considered in rapidly enlarging peri-gastrointestinal tumors with bowel obstruction. This case highlights the difficulty not only in differentiating DTF from GIST but also in accurately identifying the organ of origin.

## Abbreviations

α-SMA
: smooth muscle alpha- actin
CD34
: cluster differentiation 34
CD117
: c-kit
CECT
: contrast-enhanced CT
DTF
: desmoid-type fibromatosis
EUS-FNA
: endoscopic US-guided fine-needle aspiration
FEEA
: functional end-to-end anastomosis
GIST
: gastrointestinal stromal tumor
H&E
: hematoxylin and eosin
ICG
: indocyanine green
ISGPS
: International Study Group of Pancreatic Surgery
POPF
: postoperative pancreatic fistula

## INTRODUCTION

DTF is a rare fibroblastic neoplasm characterized by locally aggressive growth without metastatic potential.^1,2)^ Intra-abdominal DTF typically arises from the mesentery or small bowel wall and may cause severe morbidity by compressing or invading adjacent organs.^3)^ Preoperative differentiation between DTF and GIST is often challenging because of overlapping radiological and pathological features.

Here, we present a case of jejunal DTF initially diagnosed as gastric GIST. Although the tumor was considered to originate from the stomach on preoperative evaluation and during surgery, histopathological examination ultimately demonstrated its origin in the proximal jejunum. This case illustrates the difficulty of identifying the true organ of origin of an infiltrative intra-abdominal tumor and highlights the importance of careful pathological evaluation in determining its primary site.

## CASE PRESENTATION

A 56-year-old woman was referred to our hospital after an abdominal mass was incidentally detected during a health screening. The patient’s medical and surgical histories were unremarkable, and there was no family history of familial adenomatous polyposis.

CECT revealed a 65-mm-diameter heterogeneous mass along the posterior wall of the middle part of the stomach. The arterial phase demonstrated intratumoral vessels, and the equilibrium phase showed diffuse enhancement (**Fig 1A**). PET-CT demonstrated fluorodeoxyglucose uptake abnormalities without distant metastasis (**Fig. 1B**). On upper gastrointestinal endoscopy, an extraluminal mass was observed to be compressing the gastric body; however, the overlying mucosa appeared intact, with no significant abnormalities (**Fig. 1C**).

**Fig. 1:**
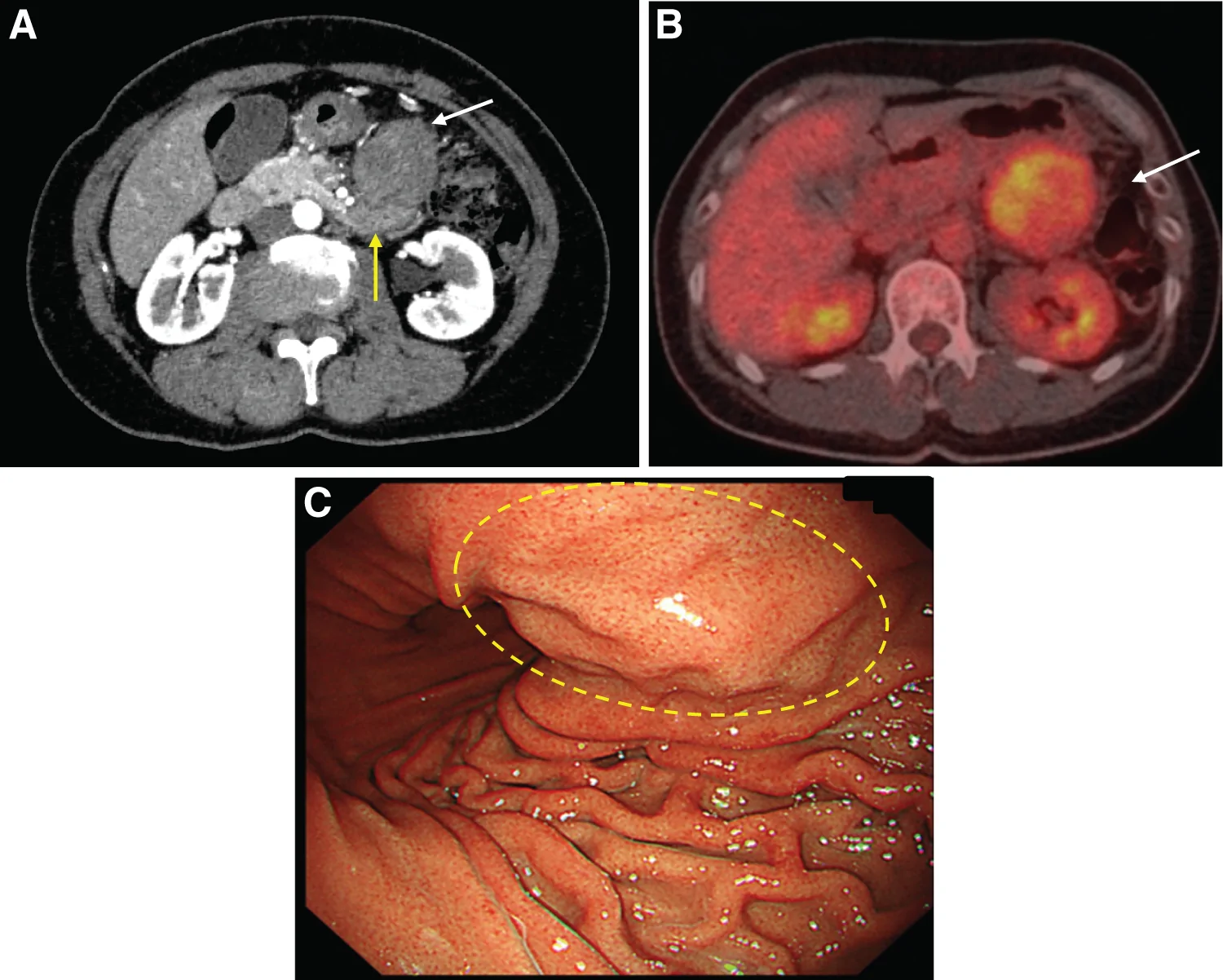
CECT on initial presentation. (**A**) CECT showing the tumor (white arrow) and duodenum (yellow arrow). (**B**) PET-CT showing abnormal uptake in the tumor (indicated by the white arrow). (**C**) Esophagogastroduodenoscopy showed extrinsic compression of the stomach due to the tumor (indicated by the dashed circle). CECT, contrast-enhanced CT

Endoscopic US demonstrated a well-demarcated hypoechoic mass arising from the fourth layer of the gastric wall. A biopsy performed at the referring hospital revealed spindle cells. Immunohistochemistry showed partial positivity for c-kit and DOG1 and negativity for S-100 and desmin. The pathological report indicated a suspicion of GIST. Surgery was scheduled approximately 3 months later according to the patient’s preference.

Shortly before the planned surgery, 3 months after the initial CT examination, the patient developed loss of appetite and vomiting. Emergency CECT demonstrated rapid tumor enlargement from 65 to 83 mm, with encasement of the proximal jejunum and compression of the duodenum (**Fig. 2A**). An upper gastrointestinal series revealed marked gastric dilatation and obstruction at the duodenum (**Fig. 2B**), and surgical intervention was performed.

**Fig. 2:**
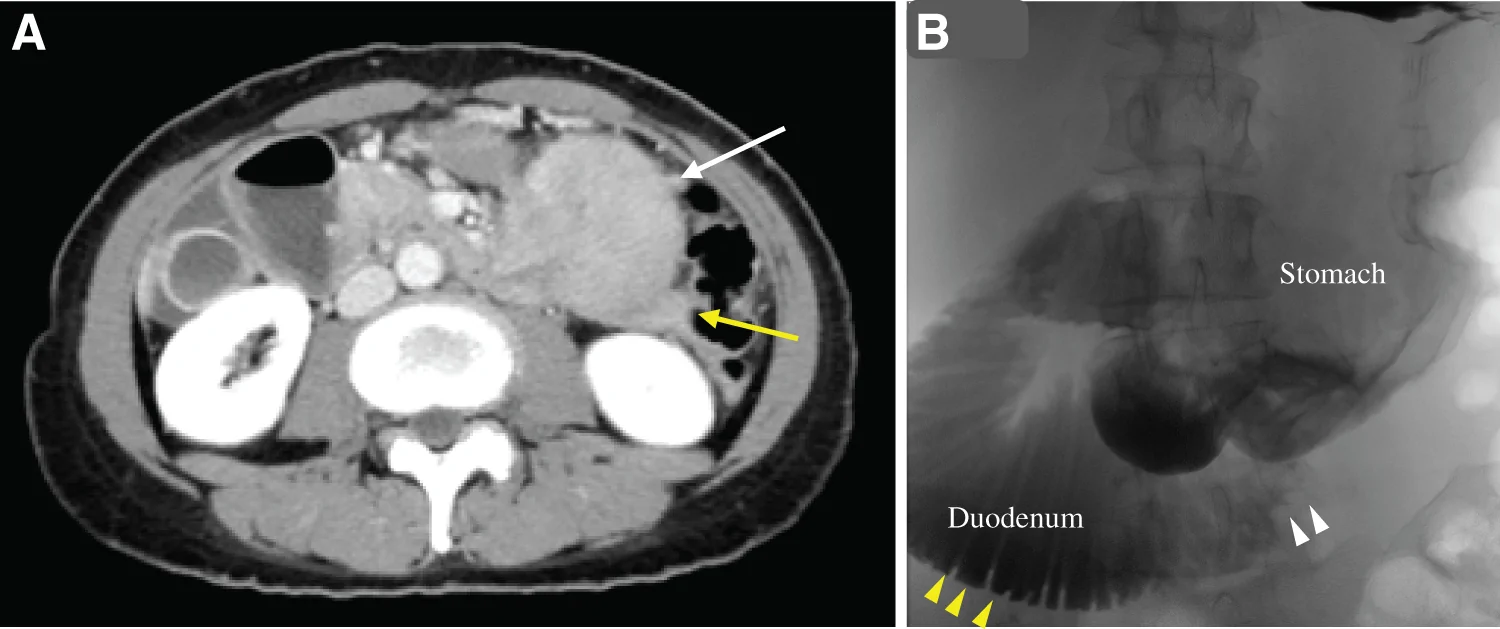
CECT and upper gastrointestinal series on emergency admission. (**A**) The tumor (indicated by the white arrow) invaded the jejunum (indicated by the yellow arrow). (**B**) The horizontal part of the duodenum shows substantial dilation (yellow triangles). There is abrupt contrast flow, with no passage of the contrast medium distal to the obstruction site (indicated by the white triangles). CECT, contrast-enhanced CT

Intraoperatively, the tumor extensively involved the posterior wall of the middle gastric body, the proximal jejunum approximately 10 cm distal to the ligament of Treitz, the transverse mesocolon, and the pancreatic body. Because of the extensive involvement of the posterior gastric wall, the tumor was considered to originate from the stomach (**Fig. 3A** and **3B**). ICG fluorescence imaging demonstrated poor perfusion of the transverse colon, consistent with the macroscopic findings, and partial colectomy was therefore performed. The jejunum and stomach were resected en bloc with the tumor. Tumor invasion into the pancreatic body also required partial pancreatectomy (**Fig. 3C**). After compressing the pancreas using an intestinal clamp for 10 minutes, it was divided using a reinforced stapler, and a polyglycolic acid sheet was applied to the pancreatic stump. Reconstruction was completed with FEEA of both the jejunum and transverse colon.

**Fig. 3:**
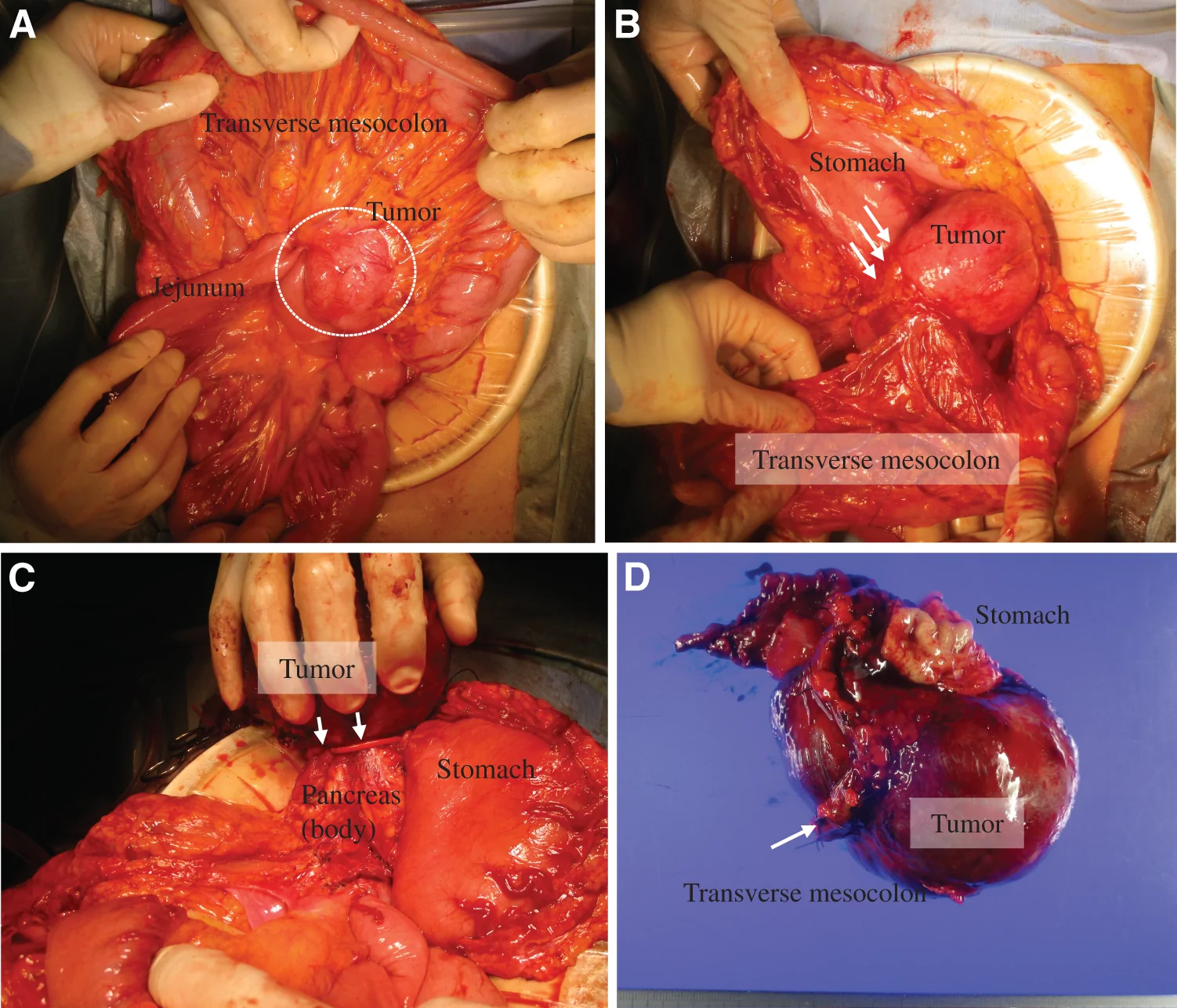
Intraoperative images and resected specimen. (**A**) Direct invasion of the transverse mesocolon (dashed circle: tumor). (**B**) The tumor is in contact with the posterior wall of the gastric body (indicated by the white arrows). (**C**) The tumor shows involvement of the pancreatic body (indicated by the white arrows). (**D**) Resected specimen and pancreatic transection line (white arrow).

The tumor measured 94 × 17 × 14 mm (**Fig. 3D**). Immunohistochemistry showed nuclear β-catenin positivity (partial), LEF1 (partial positivity), desmin (partial positivity), α-SMA (partial positivity), CD117 (negative), DOG1 (negative), CD34 (negative), S-100 (negative), and MIB-1 index 8% (**Fig. 4A** and **4B**, **Supplementary Fig. 1**). In the stomach, tumor invasion was limited to the serosa and the outer muscularis propria (**Fig. 4C**), without continuity to the gastric mucosa or submucosa. In the jejunum, tumor bundles extended from the submucosa to the subserosa, with disruption of the muscularis propria (**Fig. 4D**). The resected margins were negative. The final diagnosis was jejunal-origin DTF with secondary gastric invasion. CTNNB1 mutation analysis was not performed. Postoperatively, drain amylase levels were routinely measured on PODs 1 and 3 (4269 and 339 U/L, respectively). Serum amylase on POD 3 was 55 U/L. An upper gastrointestinal contrast study on POD 7 and CECT on POD 5 showed no evidence of anastomotic leakage. Although the drain amylase level was elevated on POD 1, it decreased substantially by POD 3, and no clinically relevant POPF was diagnosed. The patient subsequently developed an intra-abdominal abscess, classified as an organ/space surgical site infection and Clavien–Dindo grade IIIa. The exact source of the abscess could not be definitively identified. Because adequate drainage could not be achieved through the surgically placed drain, CT-guided percutaneous drainage was performed by an interventional radiologist. Culture of the drained fluid yielded AmpC-producing *Escherichia coli*, and antimicrobial therapy was changed to cefepime plus metronidazole according to the culture and susceptibility results. The patient showed a favorable clinical response, and antimicrobial therapy was discontinued after 5 days. She was discharged on POD 25. No recurrence was observed 12 months after surgery.

**Fig. 4:**
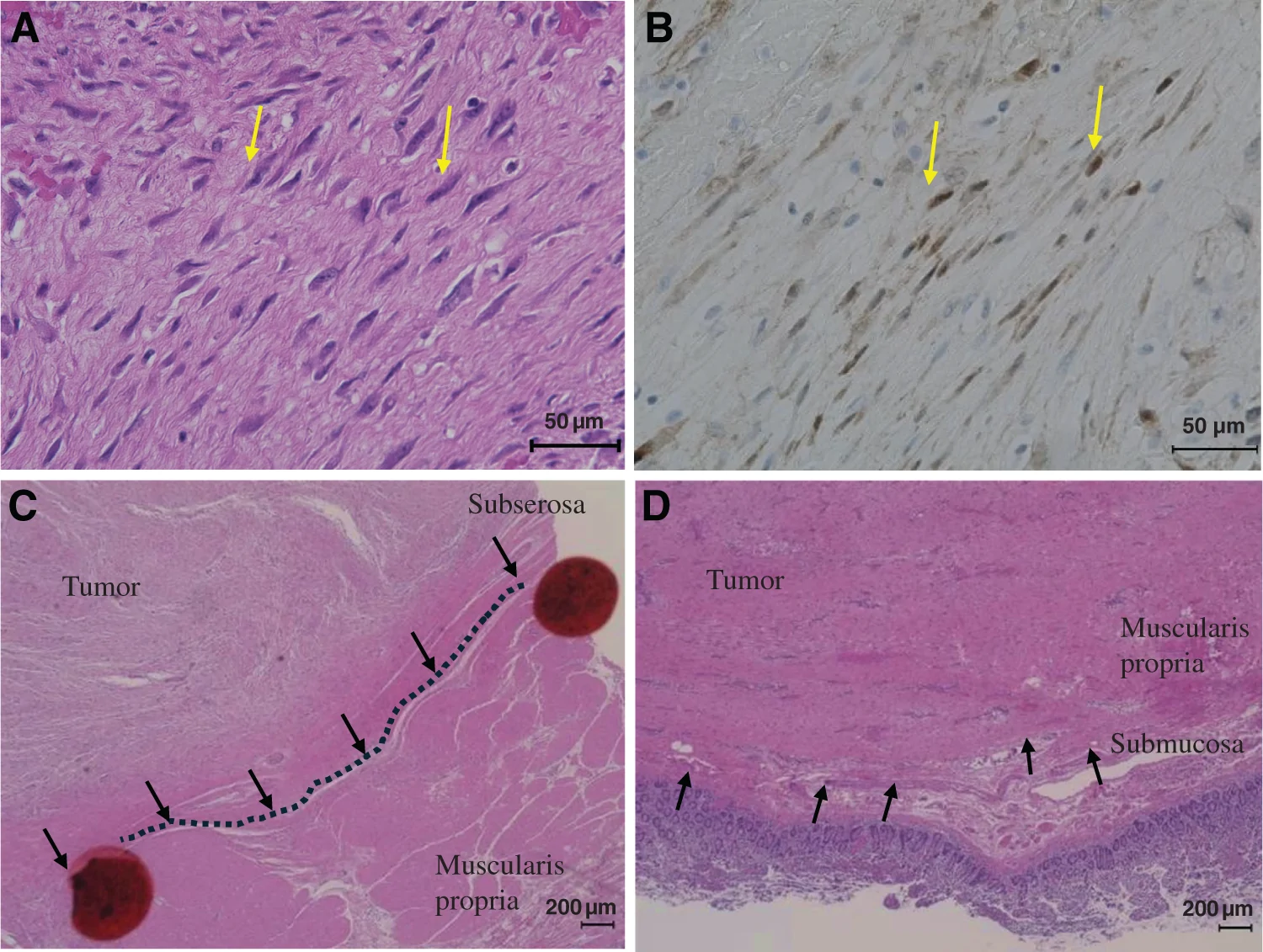
Histopathological findings. (**A**) H&E staining. Spindle cells are proliferating (indicated by the yellow arrows). (**B**) β-catenin staining. Yellow arrows show nuclear translocation in spindle cells. (**C**) H&E staining of gastric mucosa. Tumor cell proliferation is observed in the subserosa, extending to the lower border of the muscularis propria (indicated by the black arrows). (**D**) H&E staining of the small intestinal mucosa. Tumor cells grow from the submucosa to the subserosa, with disruption of the muscularis propria (indicated by the black arrows). H&E, hematoxylin and eosin

## DISCUSSION

DTF is a rare fibroblastic tumor characterized by locally aggressive growth without metastatic potential. Desmoid tumors are anatomically classified as extra-abdominal, abdominal wall, and intra-abdominal types.^4,5)^ Intra-abdominal DTF accounts for approximately 10%–15% of desmoid tumors and most commonly arises from the mesentery, whereas primary involvement of the small intestinal wall itself is uncommon.^1,6)^ Its infiltrative growth and nonspecific radiological appearance can make both histological diagnosis and identification of the primary site challenging.

The most distinctive feature of the present case was the difficulty in determining the true organ of origin. Initial CT and EUS suggested a gastric origin. Even during surgery, extensive involvement of the posterior gastric wall suggested a gastric origin. Histopathological examination ultimately demonstrated tumor involvement extending from the submucosa to the subserosa of the jejunum, with disruption of the muscularis propria, whereas gastric involvement was predominantly limited to the outer wall. The extensive involvement of the stomach, jejunum, pancreas, and transverse mesocolon likely obscured the true anatomical origin. Thus, in highly infiltrative intra-abdominal tumors involving multiple adjacent organs, careful pathological evaluation of the pattern of mural involvement is essential for determining the primary site.

Preoperative differentiation from GIST was another diagnostic challenge. The EUS-FNA specimen obtained at the referring hospital showed focal positivity for KIT and DOG1, whereas both markers were negative in the resected specimen. The limited amount of biopsy tissue may have introduced sampling bias, while differences in staining procedures between institutions may also have contributed to the discrepant results. Despite only focal nuclear β-catenin expression, the diagnosis of DTF was based on the characteristic proliferation of bland spindle cells with an infiltrative growth pattern and the overall immunohistochemical profile. The absence of KIT and DOG1 expression in the resected specimen, together with the morphological and immunohistochemical findings, also helped distinguish DTF from other spindle cell tumors, including GIST, schwannoma, and leiomyogenic tumors. CTNNB1 mutation analysis was not performed.

Another notable feature was the rapid tumor progression with bowel obstruction. DTF is often regarded as a slow-growing tumor, but its clinical course is unpredictable. Some intra-abdominal lesions demonstrate rapid enlargement and aggressive local invasion.^7,8)^ Although GIST can occasionally present with bowel obstruction, this presentation is relatively uncommon. Most GISTs exhibit exophytic growth and are more commonly associated with gastrointestinal bleeding or nonspecific abdominal pain than with mechanical obstruction.^9,10)^ Previous cases of small intestinal or mesenteric DTF presenting with duodenal or intestinal obstruction have also been reported.^11)^ Therefore, the presence of significant bowel obstruction in our patient also raised clinical suspicion that the tumor might not represent a typical GIST.

Previous intra-abdominal cases of DTF have been misdiagnosed as GIST and have occasionally presented with bowel obstruction or adjacent invasion.^3,8,11–16)^(**Table 1**)

**Table 1:** Summary of previously reported cases and the present case

Author (year)	Age/sex	Initial diagnosis	Presumed origin	Final pathological origin	Bowel obstruction	Adjacent organ involvement	Surgical procedure	Outcome
Colombo (2005)^11)^	50/M	GIST	Small bowel/mesentery	Small bowel	No	Small bowel/mesentery	Segmental small bowel resection	No recurrence
Wronski (2011)^12)^	44/F	GIST	Mesentery/small bowel	Small bowel mesentery	No	Small bowel	Segmental small bowel resection	No recurrence
Akbulut (2018)^13)^	46/F	Intra-abdominal tumor	Duodenum	Duodenum	Yes	Proximal jejunum, distal pancreas	En block resection of the duodenum, proximal jejunum, distal pancreas, and the spleen	No recurrence
Eswaravaka (2021)^3)^	31/M	GIST	Jejunum	Ileum	No	No	Segmental small bowel resection	Not reported
Michaelides (2022)^14)^	74/M	Intra-abdominal/mesenteric tumor	Small bowel mesentery	Small bowel mesentery	Yes	Adjacent small bowel	Right hemicolectomy and small bowel resection	No recurrence
Xie (2023)^15)^	31/M	Small bowel tumor	Distal ileum	Distal ileum	No	Cecum/adjacent intestine	Ileal and ileocecal resection	No recurrence
Huang (2024)^8)^	52/M	Duodenal stricture/mesenteric tumor	Jejunal mesentery	Jejunal mesentery	No	Distal duodenum	En bloc resection of jejunum and duodenum	Not reported
Nakao (2025)^16)^	69/M	Small bowel tumor	Small bowel	Small intestinal muscularis propria	No	No	Partial jejunal resection	No recurrence
Present case	56/F	Gastric GIST	Stomach	Proximal jejunum	Yes	Stomach, pancreas, and transverse mesocolon/colon	Partial jejunal resection, partial gastrectomy, partial pancreatic resection, and partial colectomy	No recurrence

F, female; GIST, gastrointestinal stromal tumor; M, male

The present case was unusual because the presumed organ of origin differed throughout the diagnostic process. This case illustrates that the infiltrative nature of DTF may obscure not only its pathological diagnosis but also its true organ of origin.

In the present case, en bloc resection was required to achieve an R0 resection due to invasive involvement of adjacent organs. Because the tumor involved the transverse mesocolon and a major marginal vein, ICG fluorescence imaging was used to assess colonic perfusion and confirmed impaired blood flow in the macroscopically discolored segment. Ten mg of ICG was administered intravenously, and near-infrared fluorescence imaging was performed. According to our institutional protocol, failure to visualize fluorescence of the bowel wall within 60 seconds after ICG administration was considered indicative of impaired perfusion. Partial pancreatectomy was also unavoidable, and prolonged pre-firing compression before stapled transection has been reported to reduce POPF.^17,18)^ Postoperative drain management was performed in accordance with the International Study Group of Pancreatic Surgery recommendations.^19)^ Although the patient developed an intra-abdominal abscess, no clinically relevant POPF (Grade B or C according to the 2016 ISGPS definition) was diagnosed.

This report has several limitations. CTNNB1 mutation analysis was not performed, and the follow-up period of approximately 12 months was relatively short. Longer follow-up is necessary because of the potential for late local recurrence.

## CONCLUSIONS

We report a case of jejunal DTF initially suspected to be a gastric GIST. Rapid tumor growth and bowel obstruction may serve as important clinical clues. Prompt surgical intervention is required for symptomatic cases, and careful follow-up is warranted due to the risk of local recurrence.

## SUPPLEMENTARY MATERIALS

Supplementary Fig. 1 Immunohistochemical staining of the tumor tissue. (**A**) The tumor cells are negative for CD34 (indicated by the yellow arrow). (**B**) Immunoreactivity for CD117 (c-kit) was also negative (indicated by the yellow arrow). (**C**) Staining for desmin was partially positive within the tumor tissue (the yellow arrow indicates the negative area). (**D**) The tumor spindle cells are partially positive for LEF-1 (the yellow arrow indicates the negative area).

## DECLARATIONS

### Funding

The authors received no specific funding for this work.

### Authors’ contributions

CI wrote the manuscript with the support of KNi and TK.

HI, KK, KT, HKo, JK, TI, KNa, HS, TA, YY, HKu, RM, HF, and AS analyzed and interpreted the patient data and supervised the research.

All authors have read and approved the final version of the manuscript.

### Availability of data and materials

The datasets used or analyzed in the current study are available from the corresponding author upon reasonable request.

### Ethics approval and consent to participate

Ethics review is not required for the publication of case reports at our institution.

### Consent for publication

Informed consent to publish this case report has been obtained.

### Competing Interests

The authors have no conflicts of interest to disclose.
