# Supplementary material for: Desmoid-Type Fibromatosis of the Proximal Jejunum Mimicking Gastric Gastrointestinal Stromal Tumor: Diagnostic Pitfalls in Determining the Tumor Origin
Source: Surg Case Rep. 2026 Oct 1;12(1):26-0440. doi: 10.70352/scrj.cr.26-0440 (PMC13634659; doi:10.70352/scrj.cr.26-0440)
Supplement: Supplementary file 1 [file scr-12-01-26-0440-s001.pdf]

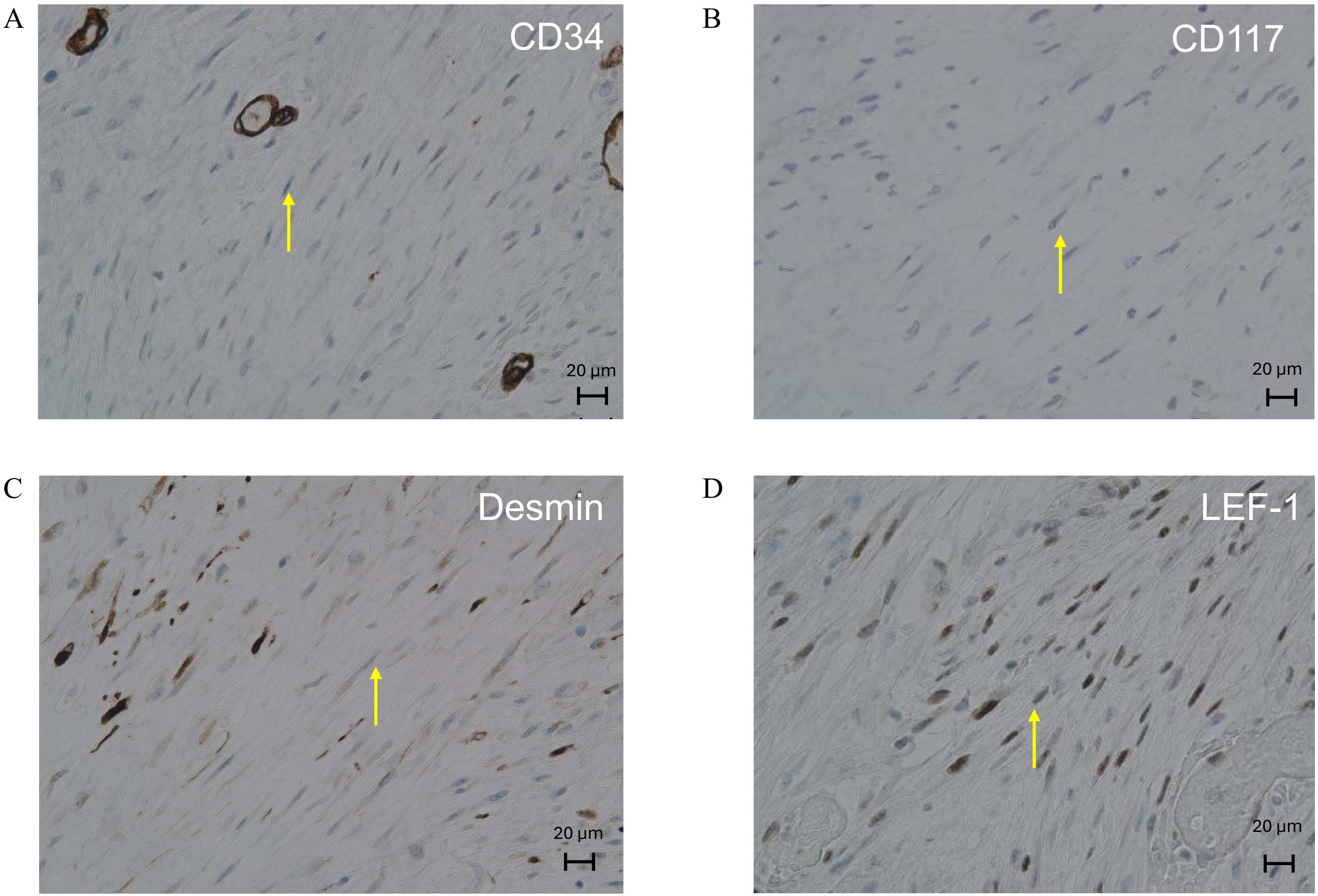
A
CD34
20 µm
B
CD117
20 µm
C
Desmin
20 µm
D
LEF-1
20 µm
